# Laparoscopic cholecystectomy for left-sided gall bladder in situs inversus totalis patient, a technically demanding procedure

**DOI:** 10.1016/j.amsu.2019.09.002

**Published:** 2019-09-11

**Authors:** Ayad Ahmad Mohammed, Sardar Hassan Arif

**Affiliations:** Department of Surgery, College of Medicine University of Duhok, DUHOK, Kurdistan Region, Iraq

**Keywords:** Laparoscopic cholecystectomy, Situs inversus, Gall stones, Left-sided gall bladder, MRCP

## Abstract

Situs inversus is a condition in which the anatomical viscera are placed in a reverse anatomical location, it may be partial affecting the thoracic organs or the abdominal organs, or total affecting both.

A 28-year-old man who was a known case of situs inversus totalis presented with epigastric pain and left hypochondrial pain. ultrasound revealed multiple gall stones in a left-sided gall bladder, laparoscopic cholecystectomy was done successfully for him with no complications.

Patient position during surgery and the sites of the laparoscopic ports greatly affect the performance during surgery. Identification of the anatomical structures which are arranged in a mirror image pattern is the key for successful surgery. The critical view of safety should be identified before any structure is clipped or divided.

A right-handed surgeon will feel more impairment during surgery for a left-sided gall bladder, while a left-handed surgeon will do it with better comfort. Surgeries for such cases are better to be performed by a surgeon with massive experience in laparoscopy or by a hepatobiliary surgeon, it may not be suitable for surgeons in training. MRCP will show the biliary anatomy prior to surgery and CT-scan with dual phase contrast will help to show any associated vascular abnormalities.

## Highlights

• Gall bladder stones are commonly diagnosed and many patients need surgical intervention.
• Situs inversus patients have a mirror image anatomy.
• A left-handed surgeon can perform laparoscopic cholecystectomy with more comfort than a right-handed one.

## 1 Introduction

Situs inversus is a condition in which the anatomical viscera are placed in a reverse anatomical location, it is either partial affecting the thoracic organs or the abdominal organs, or total affecting both. It occurs in 1/20000 live births. Situs inversus is not regarded as pathological but it may be associated with some congenital anomalies which may be life threatening [1].

Gall stones are common that may cause a variety of presentations, many cases are asymptomatic and discovered during investigations, while others may have symptoms or complications of gall stones. Laparoscopic cholecystectomy is one of the most widely performed operations worldwide [2].

Normally the gall bladder is attached to the right lobe of the liver, in some rare occasions it is located in ectopic sites such as the left side, the midline, or in other locations. In patients with situs inversus the anatomical organs are located in the reverse site, the gall bladder is located in the left upper abdominal quadrant [3].

When the patient is known to have situs inversus, the diagnosis is made easily when the ultrasound will reveal the stones in a left-sided gall bladder. CT scan or chest X-ray will show an inverse location of other organs such as the heart, lobes of the liver, spleen, etc. [1].

The aim of presenting this case is to concentrate of three points; the first one is that patients with abnormally sited gall bladder will have pain in atypical sites depending on the location of the gall bladder, the second point is that the surgeon should modify the sites of the laparoscopic ports or the site of the surgical incision in order to be able to do cholecystectomy successfully, and the third point is that laparoscopic cholecystectomy for such patients is a technically demanding procedure and it would be easier for the left handed surgeons.

Patients with left-sided gall bladder feel the pain of gall bladder diseases in the left hypochonrdium or the epigastric region, the pain may also radiate to the left shoulder. This may cause diagnostic difficulties and may be misdiagnosed as pain arising from the stomach especially in patients who are not known to have situs inversus [3].

The work in this case report has been reported in line with the SCARE 2018 criteria [4].

## 2 Patients information

### 2.1 Clinical findings

A 28-year-old man who was a known case of situs inversus presented with repeated attacks of epigastric pain and left hypochondrial pain for one year. The pain intensity was increased over a period of 2 months.

The family history was positive for situs inversus totalis, one nephew of the patient had the same malformation. There was no history of chronic drug usage and the psychosocial history was non relevant.

### 2.2 Diagnostic assessment

**Fig. 1:**
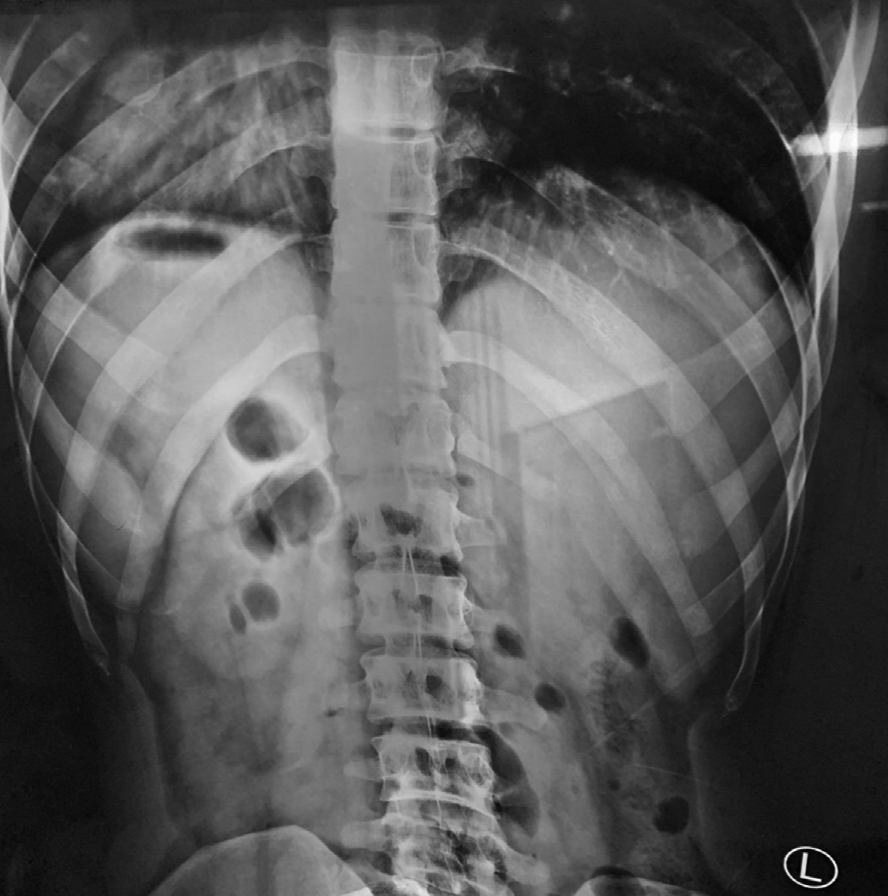
Plain X-ray of the chest and the abdomen showing the cardiac shadow in the right side of the chest and the gastric bubble under the right dome of diaphragm.

**Fig. 2:**
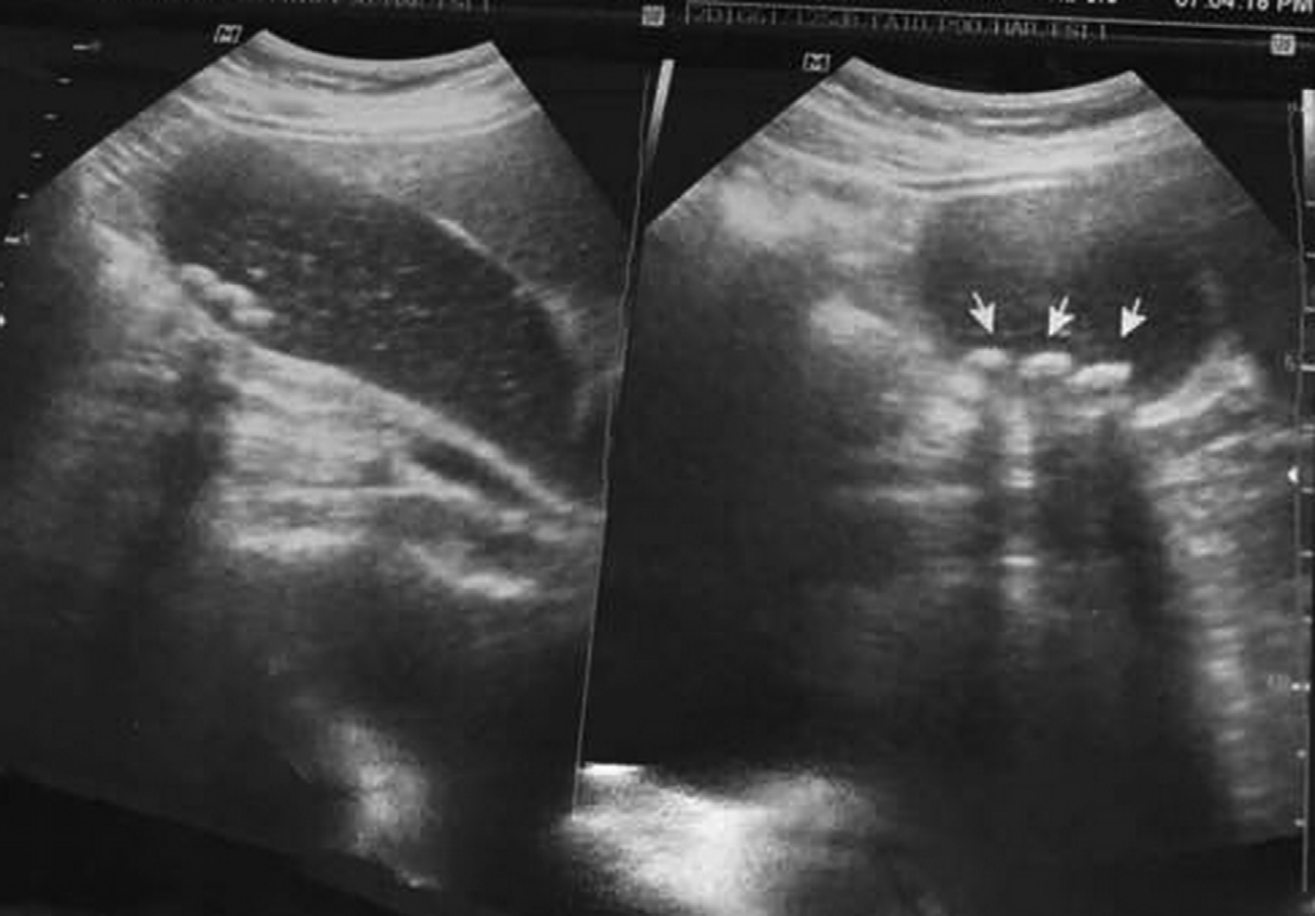
A sonographic picture showing the gall bladder containing multiple stones with no signs of acute inflammation.

Ultrasound examination was performed and revealed an evidence of multiple gall stones in a left-sided gall bladder with no signs of acute inflammation. Plain X-ray of the chest and abdomen revealed the right side location of the heart and the gastric bubble under the right dome of the diaphragm. Fig. 1, Fig. 2.

### 2.3 Therapeutic intervention

**Fig. 3:**
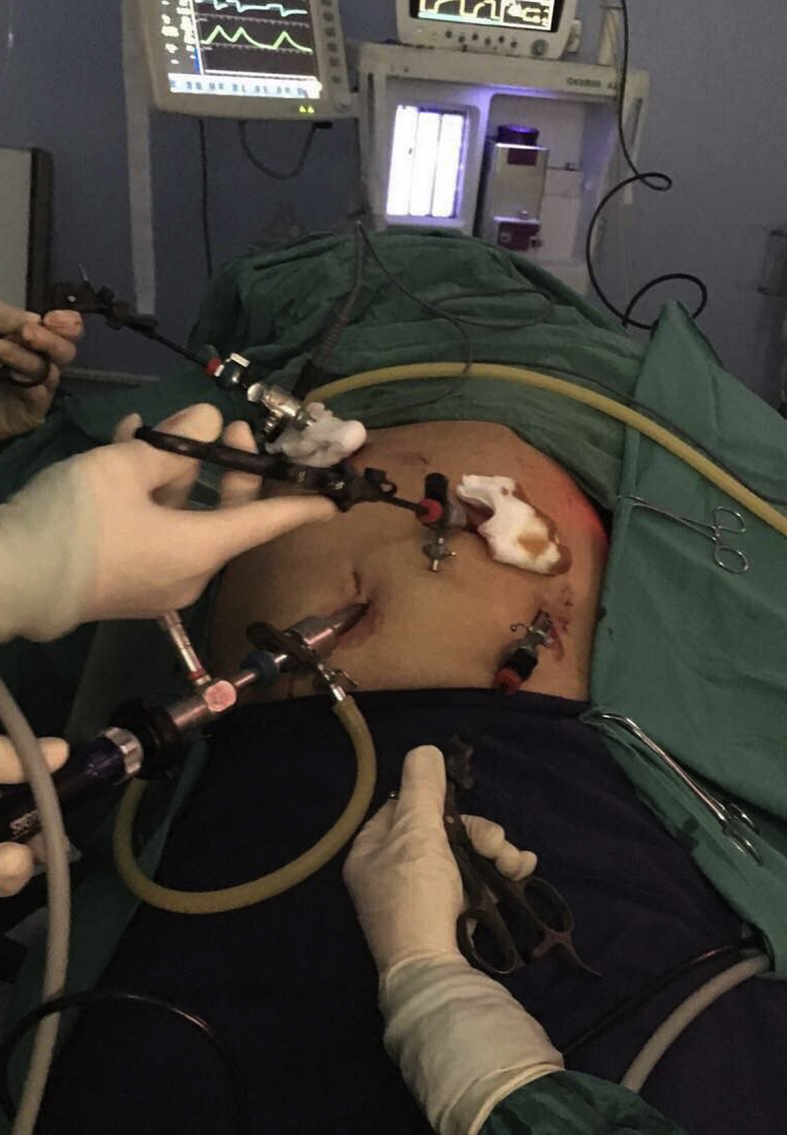
An intraoperative picture showing the placement of the laparoscopic ports, two 10 mm ports in the umbilical and the sub-xiphoidal regions, and two 5 mm ports in the subcostal region in the midclavicular and the anterior axillary lines.

**Fig. 4:**
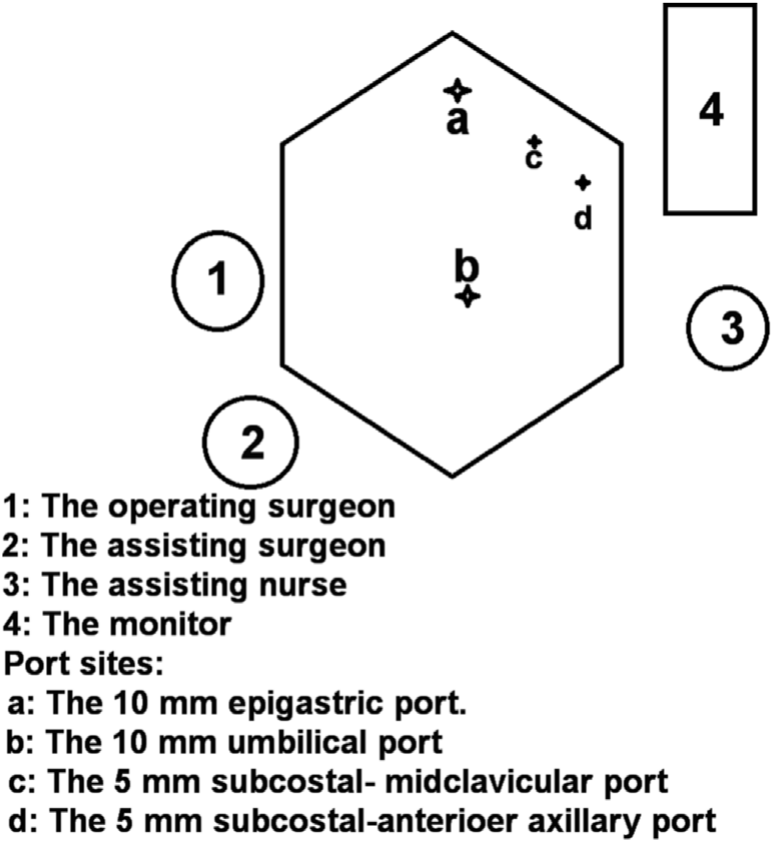
A schematic picture showing the locations of the port sites, the operating surgeon and assistants, and the location of the monitor during surgery.

During laparoscopic cholecystectomy the laparoscopic device was placed to left side of the patient instead of being in the right side in patients with normally placed gall bladder, two 10 mm laparoscopic ports were placed in the umbilical region and the epigastrium just below the xiphoidal process, the peritoneal cavity entered to the left of the falciform ligament, the other two 5 mm ports were placed in the left subcostal region in the midclavicular and the anterior axillary lines respectively (in a normally sited gall bladder these 2 ports are places in the right side in the same anatomical points).Fig. 3, Fig. 4.

**Fig. 5:**
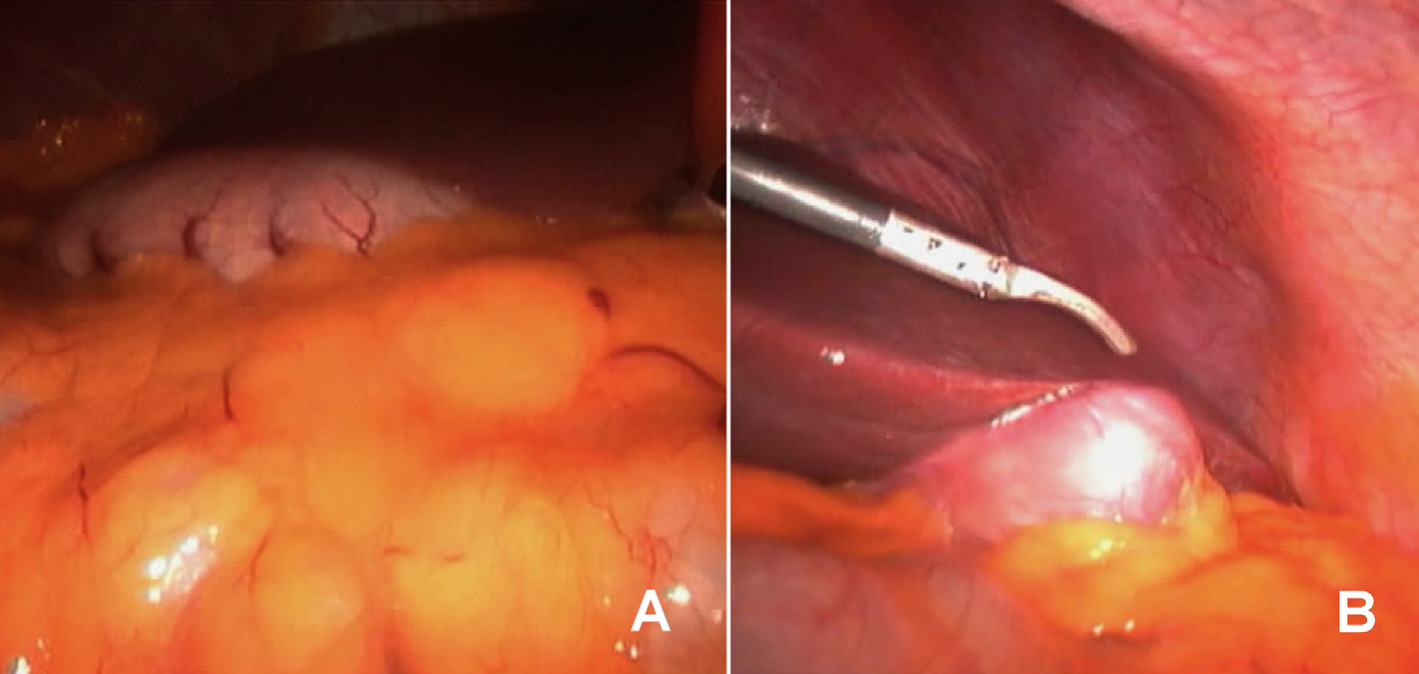
An intraoperative laparoscopic picture showing: (A) The position of the stomach in the right upper quadrant of the abdomen, and (B) The position of the gall bladder in the right left upper quadrant of the abdomen.

During surgery identification of the fundus of the gall bladder was done at the left upper quadrant and the stomach in the opposite side. Fig. 5.

**Fig. 6:**
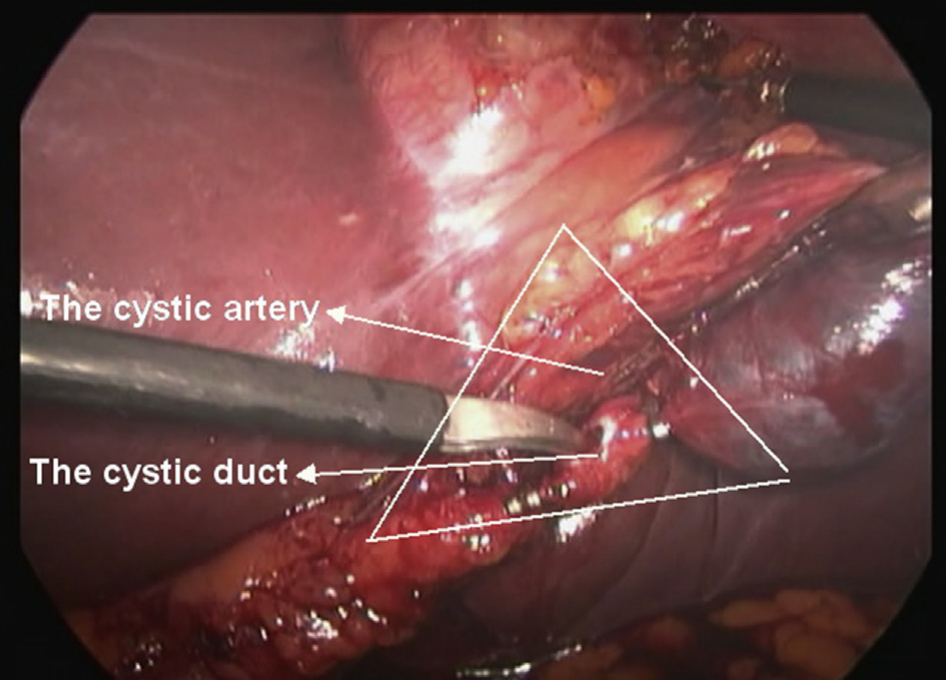
An intraoperative laparoscopic picture showing the visualization of the critical view of safety, the gall bladder remained attached to the cystic duct and the cystic artery.

Dissection of the cystic duct and artery was done and 2 proximal and 1 distal clips were applied to each of these structures. Dissection of the gallbladder containing the stones were performed and the gall bladder was extracted from the abdominal cavity with no intraoperative complications. Fig. 6.

The operation was done by 2 general surgeons who were specialized in the field of the minimally invasive and biliary surgeries, one of them was a right-handed and the other was a left-handed surgeon.

## 3 Follow-up and outcomes

No specific postoperative considerations were undertaken.

The patient was discharged next day with no postoperative complications and the 6 months follow up was uneventful.

## 4 Discussion

Anomalies of the anatomical location of the gall bladder may occur solely or in association with situs inversus, when the condition is known preoperatively the operating team will take their precautions regarding the position of the laparoscopic device, the patient position, and the laparoscopic ports sites, but the problem is that if the condition is discovered at the time of operation which will result in some confusion [3,5].

Currently there is no any clinical evidence suggesting higher incidence of gall stone in patients with situs inversus or abnormally placed gallbladder, but many authors argue in the point of higher incidence of associated biliary or vascular anomalies in such patients but no enough data are available. It is recommended that patients with such anomalies should have the anatomy of the biliary system being identified by the means of MRCP, or intraoperative cholangiography to avoid injury to the biliary system [6].

Patient position during surgery and the sites of the laparoscopic ports greatly affect the performance during surgery. The site of the laparoscopic ports for patients with situs inversus should be placed quite reversely except for the umbilical port, i.e. one 10 mm port in the sub-xiphoidal region and the peritoneal cavity should be entered to the left of the falciform ligament instead of the right in normal subjects, and two 5 mm ports placed in the subcostal region in the midclavicular and the anterior axillary lines respectively. The surgical bed during surgery should be tilted upward and to the right and the traction over the gall bladder fundus done toward the left shoulder and the Hartman's pouch toward the left iliac fossa which is the reverse in patient with normally located gall bladder [3].

Identification of the anatomical structures which are arranged in a mirror image pattern is very critical, the critical view of safety should be identified by separating the gall bladder from the gall bladder bed for 1–2 cm to identify the cystic duct and the cystic artery before the application of the clips and dividing them [3,7].

Handedness of the surgeon influences the performance during surgery, the majority of the surgical procedures practiced today are apparently designed for right-handed surgeons because of the higher percentage of right-handed population. A right-handed surgeon will feel more impairment when performing laparoscopic cholecystectomy for a left-sided gall bladder, while a left-handed surgeon will do it with better comfort [8].

### 4.1 Patient perspective

I knew that my organs are reverse and the pain at that site was due to gall bladder problem. My main concern was whether the surgery can be done using the laparoscopy or not and finally I am very happy after the surgery and I feel much better than before.

### 4.2 Informed consent

An informed written consent was taken from the patient for reporting this case and the accompanying images.

## Conflicts of interest

There is no conflict of interest.

## Funding source

The authors are the source of the funding.

### Provenance and peer review

Not commissioned, externally peer reviewed.

## Ethical approval

No ethical committee approval was needed; consent have been taken from the family to report their findings.

## Author contribution

The surgeon who performed the procedure: Dr Ayad Ahmad Mohammed and Dr Sardar Hassan Arif.

Study design, writing, and the final approval of the manuscript: Dr Ayad Ahmad Mohammed and Dr Sardar Hassan Arif.

## Guarantor

Dr Ayad Ahmad Mohammed.
